# scInterpreter: a knowledge-regularized generative model for interpretably integrating scRNA-seq data

**DOI:** 10.1186/s12859-023-05579-4

**Published:** 2023-12-16

**Authors:** Zhen-Hao Guo, Yan Wu, Siguo Wang, Qinhu Zhang, Jin-Ming Shi, Yan-Bin Wang, Zhan-Heng Chen

**Affiliations:** 1 College of Electronics and Information Engineering, Tongji University, Shanghai, 200000 China; 2 EIT Institute for Advanced Study, Ningbo, 315201 Zhejiang China; 3 Department of Endocrinology, Aviation General Hospital, Beijing, 100000 China; 4 College of Computer Science and Technology, Zhejiang University, Hangzhou, 310027 Zhejiang China; 5 Department of Clinical Anesthesiology, Faculty of Anesthesiology, Second Military Medical University / Naval Medical University, Shanghai, 200433 China; 6 Big Data and Intelligent Computing Research Center, Guangxi Academy of Science, Nanning, 530007, China

**Keywords:** Single-cell RNA-seq, Batch correction, Integration, Deep learning, Knowledge-regularized

## Abstract

**Background:**

The rapid emergence of single-cell RNA-seq (scRNA-seq) data presents remarkable opportunities for broad investigations through integration analyses. However, most integration models are black boxes that lack interpretability or are hard to train.

**Results:**

To address the above issues, we propose scInterpreter, a deep learning-based interpretable model. scInterpreter substantially outperforms other state-of-the-art (SOTA) models in multiple benchmark datasets. In addition, scInterpreter is extensible and can integrate and annotate atlas scRNA-seq data. We evaluated the robustness of scInterpreter in a variety of situations. Through comparison experiments, we found that with a knowledge prior, the training process can be significantly accelerated. Finally, we conducted interpretability analysis for each dimension (pathway) of cell representation in the embedding space.

**Conclusions:**

The results showed that the cell representations obtained by scInterpreter are full of biological significance. Through weight sorting, we found several new genes related to pathways in PBMC dataset. In general, scInterpreter is an effective and interpretable integration tool. It is expected that scInterpreter will bring great convenience to the study of single-cell transcriptomics.

**Supplementary Information:**

The online version contains supplementary material available at 10.1186/s12859-023-05579-4.

## Introduction

Single-cell RNA sequencing (scRNA-seq) is a technique that can quantitatively measure cell expression at single-cell resolution [1]. Advances in single-cell sequencing technology have greatly accelerated the identification of new cell types, the inference of gene regulatory networks, and the understanding of cell differentiation processes [2]. Single-cell sequencing has also been extensively studied in areas such as development biology [3], nervous system [4], immune system [5], and cancer therapy [6]. The efforts of single-cell Cell Atlas have resulted in massive datasets containing tens of thousands of cells, with the goal of mapping a comprehensive landscape of biological development [7] and aging [8]. Through initiatives such as the Human Cell Atlas [9], the Macaque Cell Atlas [10], the Mouse Cell Atlas [11], and the Fly Cell Atlas [12], global efforts to conduct in-depth analysis of different datasets generated by different species have greatly deepened and broadened our understanding of the activities of cell life across various species [13].

In recent years, with the rapid commercialization of single-cell technology, a large amount of scRNA-seq data has been available in public databases [14–16]. Integrating private and public data into cohort studies is a growing trend [17]. However, this process inevitably encounters batch effects, which are technical noise that can mask biological information. Effectively integrating public data from different technologies or platforms is a challenge [18]. Researchers are now able to analyze increasingly diverse and complex samples. The comprehensive analysis of these datasets from different tissues, development phases, platforms, across species provides an unprecedented opportunity to build a comprehensive landmark of cellular behavior. Three of the most fundamental questions in biology are how individual cells differentiate to form tissues, how tissues coordinate with each other, and which gene regulatory mechanisms support these processes. Single-cell sequencing technology has opened up a new way to solve the above problems. However, translating observational studies into causal mechanism models brings new challenges and requires an organic combination of theoretical, experimental and computational frameworks [19]. Therefore, an integration method that can efficiently and accurately coordinate rich data sources is critical to accelerating life science research.

We want to highlight that the rapid accumulation of single-cell datasets presents several major challenges to the integration task. Firstly, batch effects can seriously obfuscate biological information. Therefore, effective integration tools are needed to remove platform technology factors without over-correction and to preserve rare cell populations. Second, as the sample size of each dataset grows dramatically, resulting in datasets containing tens of thousands of cells, the integration method needs to be scalable. Third, different sequencing platforms have unique biases, and it is a challenge to effectively integrate the scRNA-seq data of multi-technology platforms with complex noise in real environments. Fourth, deep learning models are often considered black boxes and need to be trusted. Interpretable models can greatly alleviate this problem.

A growing number of computational tools have been proposed for integration tasks. Mutual nearest neighbor (MNN) is a batch correction method that identifies the mutual nearest neighbors between cells in different batches and uses them to adjust the expression values of cells in each batch [20]. MNN is an effective tool for removing technical factors in various scenarios. However, due to the need to compute highly variable or entire genome features, MNN cannot be applied to a large number of single-cell integration tasks. BBKNN is a graph-based data integration algorithm that performs batch correction at the neighborhood graph inference step. It is designed to align multiple types of single-cell datasets generated by multiple technologies with high efficiency and accuracy [21]. BBKNN is a method that uses features after linear dimensionality reduction, which alleviates the problem that MNN cannot handle large amounts of data. Harmony is an algorithm that projects single cells into a shared embedding space, where cells are grouped by cell type rather than by dataset-specific conditions. It corrects for batch effects and preserves dataset-specific populations [22]. Harmony is a sensitive approach to correct data across multiple platforms. However, in some cases, it can lead to over-integration. Scanorama is an algorithm that identifies and merges the shared cell types among all pairs of datasets and accurately integrates heterogeneous collections of scRNA-seq data. It is sensitive to subtle temporal changes within the same cell lineage, successfully integrating functionally similar cells across time series data [23]. Scanorama is a tool that runs on multiple cores to speed up its computation. However, it still requires a lot of memory and time to process large amounts of large cell data. Additionally, it can not interpret the meaning of each dimension in the embedded space.

To address these above challenges simultaneously, we proposed scInterpreter to align multiple types of single-cell datasets generated by multiple technologies with high efficiency and accuracy. Firstly, scInterpreter is a deep learning-based model that can process large amounts of data through mini-batch strategy. Secondly, the embedding dimension is set to the number of pathways and constrain the decoder weights by prior knowledge, which allows for the estimation and explanation of cell function based on the amount of expression in each dimension. The performance of scInterpreter has been demonstrated in multi-technology and other real scenarios through a wide range of experiments. Compared to other state-of-the-art methods, scInterpreter achieves better biological variation preservation performance during the integration process while enabling the integration and annotation of atlas-level cells. We show that scInterpreter is broadly applicable to integrating datasets across different samples, platforms, and cell types. scInterpreter is a robust and efficient integration of samples from many different platforms under the interference of multiple perceived noise. For interpretability, each dimension of the cell can represent a pathway, and through different embedding expressions, the relationship between gene-pathway-phenotype can be established to provide guidance for novel biological discovery.

## Results

### Model overview

**Fig. 1:**
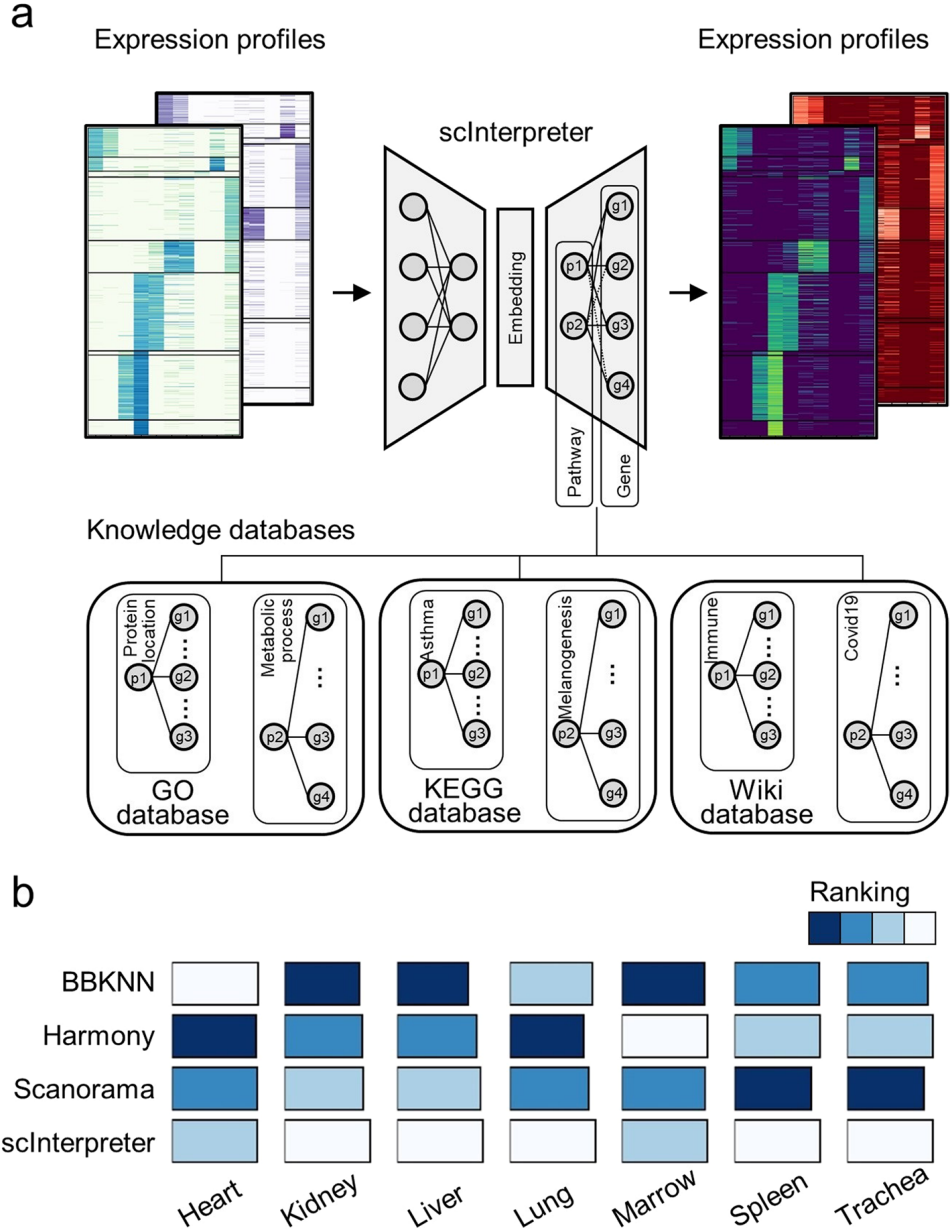
**a** model overview. **b** results of scInterpreter, BBKNN, Harmony, Scanorama in multiple tissues of the mouse atlas

Different studies that measure single cell expression have a specific batch effect, which can obscure meaningful biological information. To address this problem, we propose scinterpreter, an interpretable deep learning model that can learn the unified representation of cells in the embedding space (Fig. 1a). scInterpreter is a VAE-based generation model that consists of an Encoder and a decoder. The encoder is designed to remove the batch effects, and the generator simulates this process. We design a constraint for the weight of the decoder so that it is similar to that of the pathway-gene adjacent matrix in the knowledge database. Specifically, we treat the L1 or L2 norm distance of prior knowledge and decoder parameters as a loss function ${L}_{k}$ in the training process and optimize it (see Methods). After training, samples (cells) from the original domain can be transformed into a unified space to form a complete dataset for downstream analysis. For more details, please refer to Methods.

### Quantitative benchmarking between scInterpreter and SOTA methods

To comprehensively evaluate scInterpreter and other SOTA methods, we applied a wide range of evaluation metrics across multiple tissues of the mouse scRNA-seq atlas [24]. Specifically, BBKNN [21], Harmony [22] and Scanorama [23] were selected as the baseline methods. ARI, NMI, ASW, IsoF1 and Silhouette are adopted as the evaluation metrics. Tissues of Heart, Kidney, Liver, Lung, Marrow, Spleen and Trachea which generated by two kinds of technology including FACS [25] and Drop-seq [26, 27] are selected as the benchmark datasets.

From the results, we can find that scInterpreter achieved competitive performance compared with baselines in almost each tissue (Fig. 1b, Additional file 1: Figs. S1, S2). All methods can remove technology effects and integrate scRNA-seq data in most cases. scInterpreter has the ability to preserve the rare cell population. Specifically, in the heart dataset (Additional file 1: Fig. S3a), all methods cluster Cardiac muscle cell, endothelial cell, fibroblast, leukocyte, endocardial cell, and myofibroblast cell into isolated groups, but cannot find myofibroblast cell. In kidney dataset (Additional file 1: Fig. S3b), scInterpreter and Scanorama can separate almost all types of cells, especially they can identify endothelial cell and mesangial cell but the other two methods failed. In Spleen dataset (Additional file 1: Fig. S4a), the clusters generated by scInterpreter are tighten but the clusters generated from other 3 methods are confused together. In the Trachea dataset (Additional file 1: Fig. S4b), scInterpreter successfully aggregates epithelial cells into a single group, while other methods disperse epithelial cells into multiple clusters. The above results show that scInterpreter can not only effectively integrate scRNA-seq data from different sequencing technologies, but also can retain unique cell types in each batch.

### scInterpreter can accurately integrate and annotate whole-organism cell atlases

**Fig. 2:**
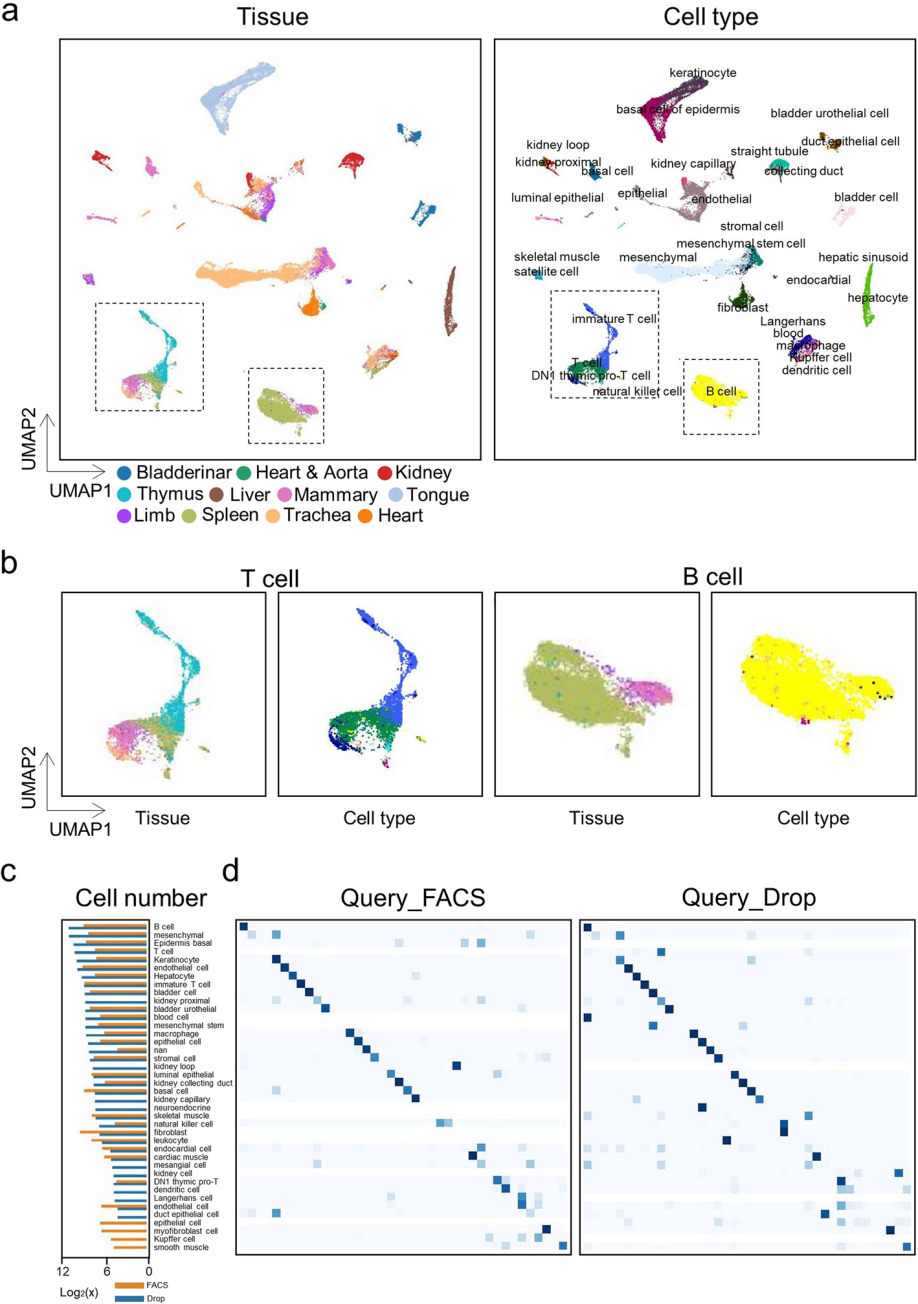
**a** we visualized the integration results from scInterpreter of mouse altas with UMAP. Cells are colored by the Tissues (the first column) and cell type (the second column). **b** UMAP embeddings of the scInterpreter results on T cell and B cell. **c** cell number statistics of mouse atlas. **d** confusion matrix of KNN classifier on scInterpretercell embedding

We designed a more challenging task where we combined all the tissues as a mouse scRNA-seq atlas. These data were generated by FACS and Drop-seq techniques. There is not only a technical confusion between the two batches, but also differences in cell types (Fig. 2c). Before integration, there was a strong batch effect between multiple datasets (Additional file 1: Fig. S5a), scInterpreter successfully removed the batch effect (Fig. 2a, Additional file 1: Fig. S5a), and integrated cells from the same type into the same clusters. T cells and B cells, common immune cells and peripheral blood cells, have been detected in many tissues. scInterpreter reasonably integrates T cells and B cells from multiple tissues together. In addition, T cell-associated subpopulations were also clustered together (Fig. 2b).

Based on the embedding generated by scInterpreter, we trained a KNN classifier to predict the cell types of Drop-seq and FACS as query by taking FACS and Drop-seq as reference, respectively. The confusion matrix of the prediction results was shown in (Fig. 2d). From the accurate cell annotation results, we can deduce that the cell representation generated by scInterpreter is reasonable. These results prove that scInterpreter is a extensible tool that can process atlas-level data, which greatly expands the scope of application of scInterpreter.

### scInterpreter can robustly integrate multi-tech scRNA-seq data

**Fig. 3:**
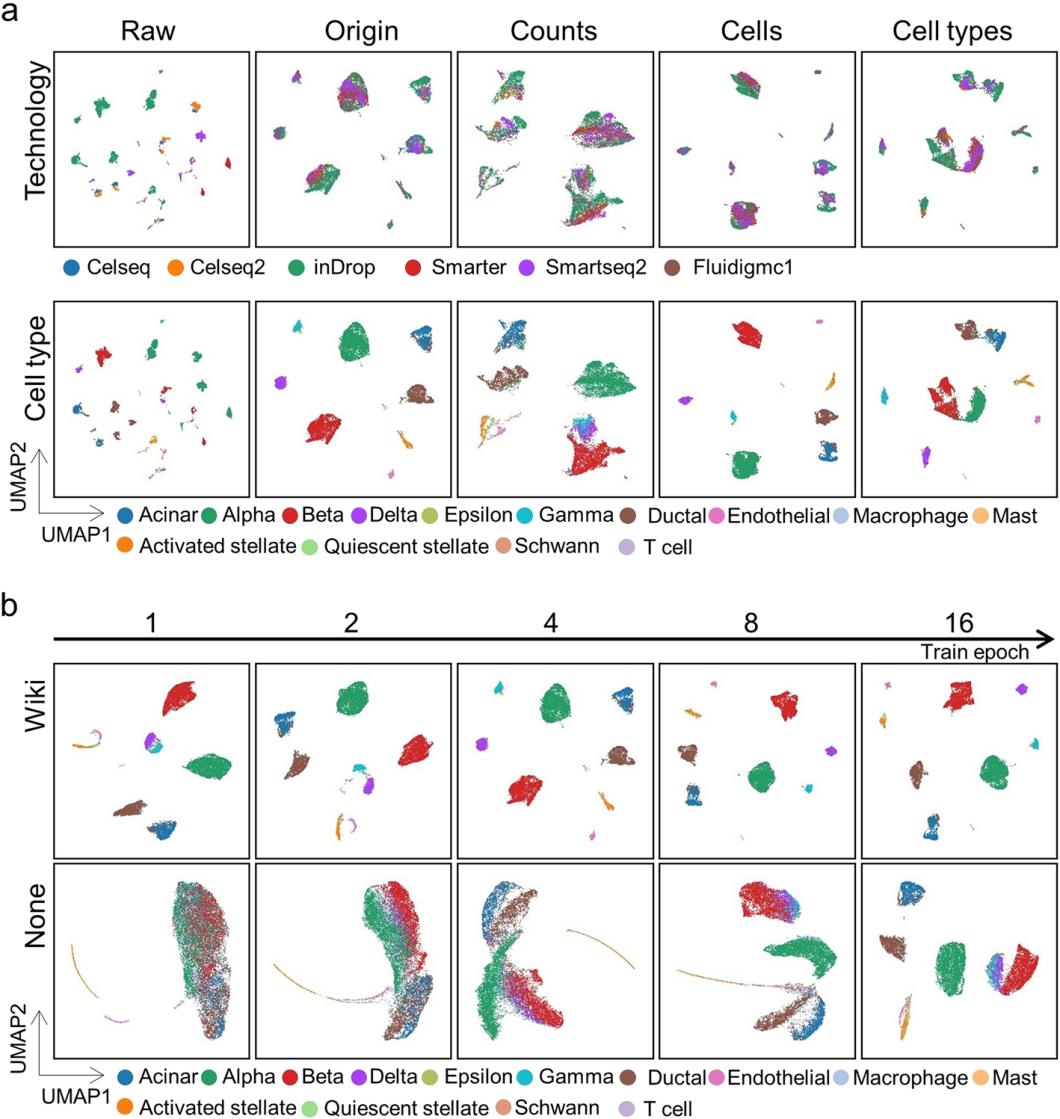
**a** we visualized the integration results from scInterpreter on the human pancreas datasets with UMAP. Cells are colored by the batch (the first row) and cell type (the second row). **b** we visualized the integration results from scInterpreter on the human pancreas datasets with UMAP. The results of with wiki (the first row) and without any knowledge database (the second row)

To further evaluate the performance of scInterpreter on multi-batch datasets, we selected a pancreas scRNA-seq dataset generated by six kinds of techniques as the benchmark [28–32]. Before integration, strong technical noise confused the biological information, and it was difficult to cluster different types of cells. After scInterpreter integration, different types of cells are clearly separated (Fig. 3a, Additional file 1: Fig. S6a).

To further evaluate the robustness of scInterpreter, we performed comparison experiments on the original data by sampling counts, sampling cells, and sampling cell types, respectively. Specifically, for sampling counts, 50% of the counts in the cell expression matrix were randomly lost. Such noise is fatal for single-cell sequencing because the expression of potential marker genes may be lost. Thus it is difficult to distinguish different types of cells. Under such conditions, scInterpreter can still clearly characterize different types of cells. BBKNN, Harmony and Scanorama confuse the cells of the endocrine glands (Alpha, Beta, Gamma, Delta) (Additional file 1: Fig. S6b). For sampling cells, 50% of the cells were randomly lost. Deep learning-based methods often require a huge amount of data to train a model to achieve excellent performance. This situation will greatly impact the performance of deep learning-based methods. But results show that scInterpreter is still easy to converge (Additional file 1: Fig. S7a). Finally, we sampled cell types. We discarded ductal, acinar, alpha, beta, delta and gamma from Celseq, Celseq2, inDrop, Smarter, Smartseq2 and Fluidigmc1 respectively. From this scenario, we evaluated the robustness of the model (Additional file 1: Fig. S7b).

### The prior knowledge accelerates the training of the scInterpreter

The prior knowledge (pathway-gene adjacency matrix) is obtained from previous wet experiments. Each pathway is connected to several associated genes, and these connected genes contribute to the functional expression of the pathway (Fig. 1a). We assume that the weight of the neural network obtained through training is similar to the pathway-gene adjacency matrix to a certain extent. Then we can infer that the training process can be accelerated through the constraints of the prior knowledge. Based on this assumption, we carried out experiments on the pancreas dataset to determine whether to add the prior knowledge constraint or not. The results showed that after adding the prior knowledge, the model converged after only one epoch, and cell types were reasonably separated after four epochs. Without the prior knowledge constraint, the training process of the model became stable after 16 epochs (Fig. 3b). We can observe that the L1/L2 loss calculation added negligible time consumption. Additionally, we recorded the time of baseline methods, including BBKNN, Harmony, and Scanorama (Table S1). We can see that BBKNN achieved the best performance in speed comparison. However, it cannot mix Alpha and Beta cells well (Additional file 1: Fig. S6a). Besides, Harmony and Scanorama achieved competitive performance, even though they took a long time.

### The cell embedding of the scInterpreter is full of interpretability

**Fig. 4:**
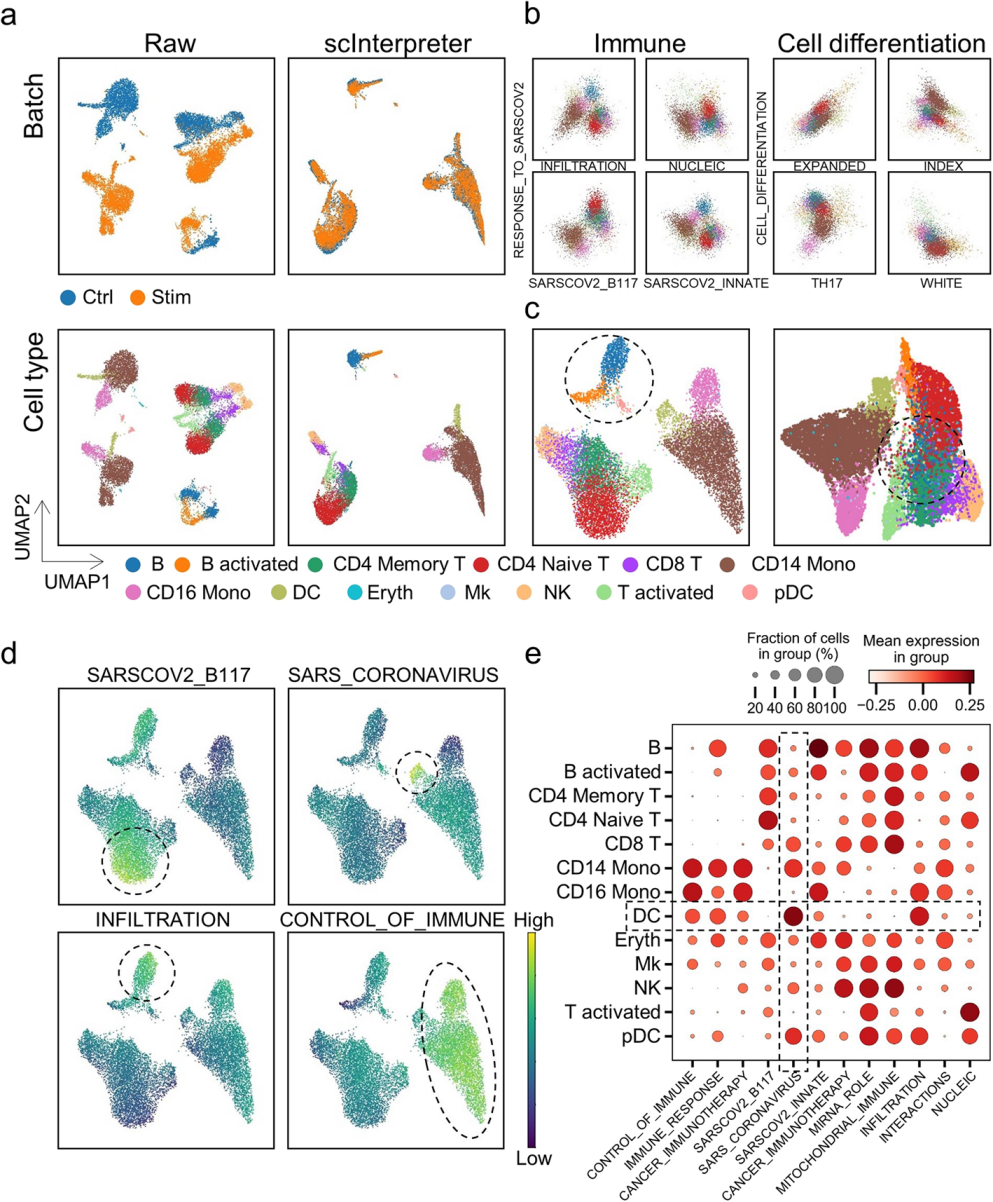
**a** we visualized the results from raw data and scInterpreter on PBMC dataset with UMAP. Cells are colored by the batch (the first row) and cell type (the second row). **b** plots of cells in 2 dimension of Immune and Cell differentiation-related pathway. **c** we visualized the integration results from scInterpreter. Cells are colored by cell type. Left is based on Immune pathway and right is based on Cell differentiation pathway. **d** UMAP plots of Immune pathway expression in cells. e. Dot plot of Immune pathway expression in different cell types

Each dimension of the cell embedding generated in bottle neck of scInterpreter represents a pathway. Through it, we can understand the phenotype and function of a cell. We performed analysis On PBMC data. There exist strong batch effects between Ctrl and Stim data before integration. After integration by using the wiki knowledge database, scInterpreter successfully eliminated the technical noise (Fig. 4a).

We found that immune-related pathways could clearly distinguish different cell types, even if only two dimensions were selected. For example, the cell type can be spited into 3 groups by using 2 pathways of INFILTRATION and RESPONSE_TO_SARSCOV2 in immune-related pathways. But when cells were represented by differentiation-related pathways, all cells were confused (Fig. 4b). Then we select and concatenate all 12 immune-related pathways in bottle neck layer as the cell representation, each cell type can be clearly grouped and displayed in the UMAP. But when we used the same method to represent cells through the cell differentiation-related pathways, B cells, B activated cells, and pDC are all confused in the T-cell-associated community (Fig. 4c). This may be because cytokine stimulation is strongly associated with immune-related pathways.

In addition, we found that different pathways are expressed differently in different cell types, and these pathways can also easily distinguish cell types (Fig. 4d, e, Additional file 1: Fig. S8a). For example, pathway SARS_CORONAVIRUS includes many IFN family genes, which are obviously associated with DC cells.

Moreover, the weight of the decoder between pathway and genes reflects the contribution of genes to modules to a certain extent. We saved the highest weights and the highest absolute weights of pathway CONTROL_OF_IMMUNE and analyzed them one by one. We found that almost all of them were immune-related [33] but not in the immune pathway dataset of the wiki database (Table S2, Table S3). This result implies that scInterpreter can not only use prior knowledge to accelerate training but also learn knowledge that does not exist in the prior database.

The pathway-gene matrix can be used as a mask in the decoder and it can also be used in the both encoder and decoder. We tested these strategies individually to get the optimal solution. From the results, it can be seen that the method that passes the l2 constraint in the decoding part is the best (Additional file 1: Figures S9a, S9b).

## Discussion

With the rapid development of single-cell sequencing technology, a large amount of single-cell data has been accumulated. It is a challenge to efficient integrate these data. Besides, how to take full use of the prior knowledge into the model to accelerate the training process and interpret the results is urgent.

Here we propose scInterpreter, a generative deep learning model that combines prior knowledge to integrate large amounts of scRNA-seq data. First, we compared scInterpreter with the state-of-the-art (SOTA) model both quantitatively (broad evaluation metrics) and qualitatively (UMAP plots) in multiple tissues of mice. scInterpreter achieved competitive performance compared with baseline methods. Secondly, we carried out atlas-level integration. ScInterpreter reasonably integrated mouse single-cell transcriptome maps and accurately annotated cell types. Then, to evaluate the performance of scInterpreter in multiple batches and under multiple conditions, we applied scInterpreter on pancreas data which composed of six techniques and various sampling manners. We found that the prior knowledge greatly accelerates the training speed and shortens the training time. Finally, we evaluated the interpretability of scInterpreter. scInterpreter is indeed able to obtain highly interpretable cell embedding and weight matrices. In summary, we believe that scInterpreter is a powerful computational tool for integrating scRNA-seq data.

However, there are still some drawbacks that need to be addressed. Firstly, selecting a suitable database for the tissue or organ under study is an important challenge. Secondly, the pathway-gene relationship in the knowledge base is often incomplete or faulty, and we need to find a more general way to incorporate prior knowledge into neural networks. The mask method makes us fully believe in the knowledge database, and the constraint of L norm makes the weight of the pathway-gene relationships all 1. Finally, we need to carefully analyze the results of predictions that conflict with prior knowledge.

## Methods

### scInterpreter model

The architecture of scInterpreter can be seen in Fig. 1a which consists of an Encoder $E$ and a Decoder $D$. The aim of $E$ is to encode sample $x$ from different batches into embedding space in a non-linear way, and the aim of $D$ is to reconstruct $x$ from the embedding representation from bottle neck layer.

The aim of scInterpreter is to maximize the log-likelihood of the observed single-cell data $\left(x\right)$

$$\mathit{log}p\left(x\right)=\mathit{log}{\int }_{z}p\left(x,z\right)dz\ge {E}_{q\left(z|x\right)}\left[\mathit{log}\frac{p\left(x,z\right)}{q\left(z|x\right)}\right]={L}_{ELBO}\left(x\right)$$

${L}_{ELBO}\left(x\right)$ can be further decomposed into two terms:

$${L}_{ELBO}\left(x\right)={E}_{q\left(z|x\right)}\left[logp\left(x|z\right)\right]-{D}_{KL}(q(z|x)||p(z))$$

The purpose of the first item is to minimize the distance between the output of decoder ${x}{\prime}$ and the input $x$. The second term is the regularization term. The purpose of it is to minimizes the Kullback-Leibeler divergence between posterior distribution $N\left(\mu ,{\sigma }^{2}\right)$ and prior distribution $N(\mathrm{0,1})$ of latent representations $z$.

In order to make the parameters of the decoder sparse and similar to the pathway-gene bipartite adjacency graph in the knowledge database, we apply the L1/L2 norm regularization to constraint the decoder:

$${L}_{k1}={\Vert {w}_{D}*\widehat{A}\Vert }_{1}$$

$${L}_{k2}={\Vert {w}_{D}*\widehat{A}\Vert }_{2}$$

where ${w}_{D}$ represents the weights of the decoder, $\widehat{A}$ represents pathway-gene adjacency matrix, * represents the Hadamard product. When training the model, we use the $\widehat{A}$ as prior information to constrain ${w}_{D}$. We assume that $\widehat{A}$ includes most of the potential relationships between pathways and genes. Given this assumption, we construct a regularization term ${L}_{k1}$ or ${L}_{k2}$ to penalize ${w}_{D}$.

### Baseline methods

Harmony: Harmony is an algorithm that projects single cells into a shared embedding space, where cells are grouped by cell type rather than by dataset-specific conditions. It corrects for batch effects and preserves dataset-specific populations.

Harmony (0.0.9) is applied in current research.

BBKNN: BBKNN is a graph-based data integration algorithm that performs batch correction at the neighborhood graph inference step. It is designed to align multiple types of single-cell datasets generated by multiple technologies with high efficiency and accuracy. BBKNN (1.5.1) is applied in current research.

Scanorama: Scanorama is an algorithm that identifies and merges the shared cell types among all pairs of datasets and accurately integrates heterogeneous collections of scRNA-seq data. It is sensitive to subtle temporal changes within the same cell lineage, successfully integrating functionally similar cells across time series data. Scanorama (1.7.3) is applied in current research.

### Integration metrics

We use a wide range of metrics including ARI, NMI, ASW, IsoF1 and Silhouette to evaluate scInterpreter and other baseline models.

ARI (Adjusted Rand Index):

ARI measures similarity by calculating the distribution of logarithmic points in the results of two clusters. The value range of ARI is [-1, 1], and the closer the value is to 1, the more similar the two clustering results are. Rand Index could be defined as follows:

$$RI=\frac{a+b}{\left(\begin{array}{c}n\\ 2\end{array}\right)}$$

where $C$ represents the true label and $K$ represents the cluster label. Define $a$ as the number of instance pairs that are divided into the same class in $C$ and the same cluster in $K$. Define $b$ as the number of instance pairs that are divided into different classes in $C$ and into different clusters in $K$.

$$ARI=\frac{RI-E(RI)}{\mathit{max}\left(RI\right)-E(RI)}$$

where $E(RI)$ represents Expectation of $RI$.

NMI (Normalized Mutual Information):

NMI measures similarity by calculating mutual information of two cluster results and performing normalization. The value range of NMI is [0, 1], and the closer the value is to 1, the more similar the two clustering results are.

$$NMI=\frac{I(P;T)}{\sqrt{H(P)H(T)}}$$

where $P$ and $T$ are categorical distributions for the predicted and real clustering, $I$ is the mutual entropy, and $H$ is the Shannon entropy.

ASW (Average Silhouette Width):

ASW evaluates respect to cell type labels, where a higher score means that cells are closer to cells of the same cell type. As ASW lies between − 1 and 1, we rescaled the score by cell type.

$$ASW=\frac{1+ASW\left(cell type\right)}{2}$$

#### IsoF1 (isolated label F1)

Isolated label F1 is developed to measure the ability of integration methods to preserve dataset-specific cell types. Isolated label F1 ranges between 0 and 1, where 1 shows that all cells of dataset-specific cell types are captured in separate clusters.

#### Silhouette

The Silhouette score is a metric used to evaluate the effect of clustering. It combines two factors, the degree of cohesion and the degree of separation, and can be used to evaluate the influence of different algorithms on the clustering results on the basis of the same original data. The value range of Silhouette score is [-1, 1].

$$silhouette score=\frac{b-a}{max(a,b)}$$

where $a$ represents the average intra-cluster distance and $b$ represents the average nearest-cluster.

### Supplementary Information

**Additional file 1**.

## Data Availability

All data used in this study are publicly available, and the details of these data can be found in Table S4. Human knowledge database can be downloaded in URL: (‘https://www.gsea-msigdb.org/gsea/msigdb/human/collections.jsp’). Mouse knowledge database can be downloaded in URL: (‘https://www.gsea-msigdb.org/gsea/msigdb/mouse/collections.jsp’). For all scRNA-seq datasets, we follow standard steps of scanpy, which are as follows. Firstly, we filter cells (scanpy.pp.filter_cells(adata, min_genes=600)) and genes (sc.pp.filter_genes(adata, min_cells=3)). Then we normalize (sc.pp.normalize_total(adata)) and logarithmize the data (sc.pp.log1p(adata)) with default paraments. After that, we select top 2000 highly variable genes as the input for model (sc.pp.highly_variable_genes(adata, n_top_genes=n_top_features).

## Abbreviations

scRNA-seq: Single-cell RNA-seq
SOTA: State-of-the-art
MNN: Mutual nearest neighbor
ARI: Adjusted rand index
NMI: Normalized mutual information
ASW: Average silhouette width
IsoF1: Isolated label F1
