# Supplementary material for: scInterpreter: a knowledge-regularized generative model for interpretably integrating scRNA-seq data
Source: BMC Bioinformatics. 2023 Dec 16;24:481. doi: 10.1186/s12859-023-05579-4 (PMC10724984; doi:10.1186/s12859-023-05579-4)
Supplement: Supplementary file 1 — Additional file 1. [file 12859_2023_5579_MOESM1_ESM.pdf]

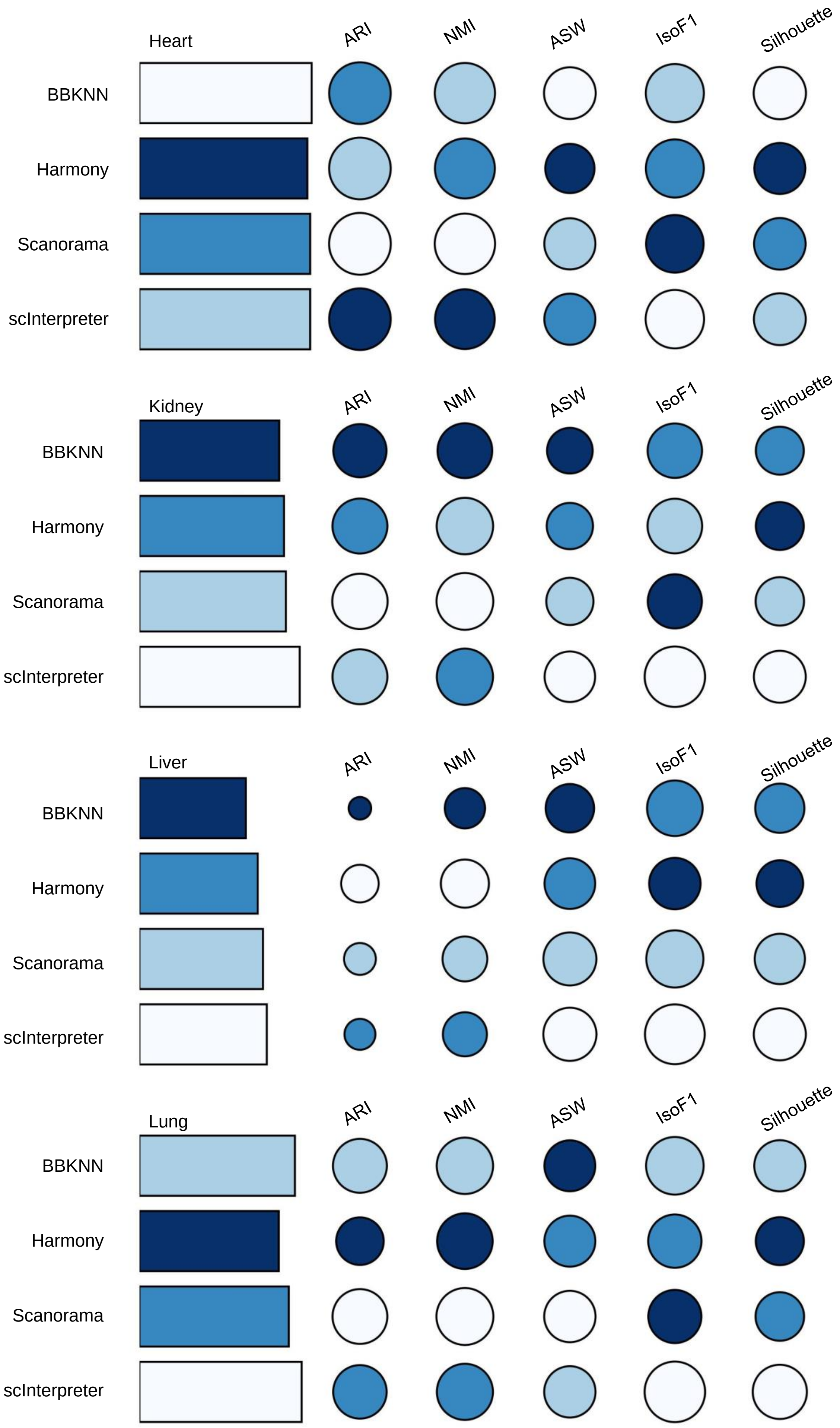

Figure S1, results of scInterpreter, BBKNN, Harmony, Scanorama in multiple tissues of the mouse scRNA-seq atlas

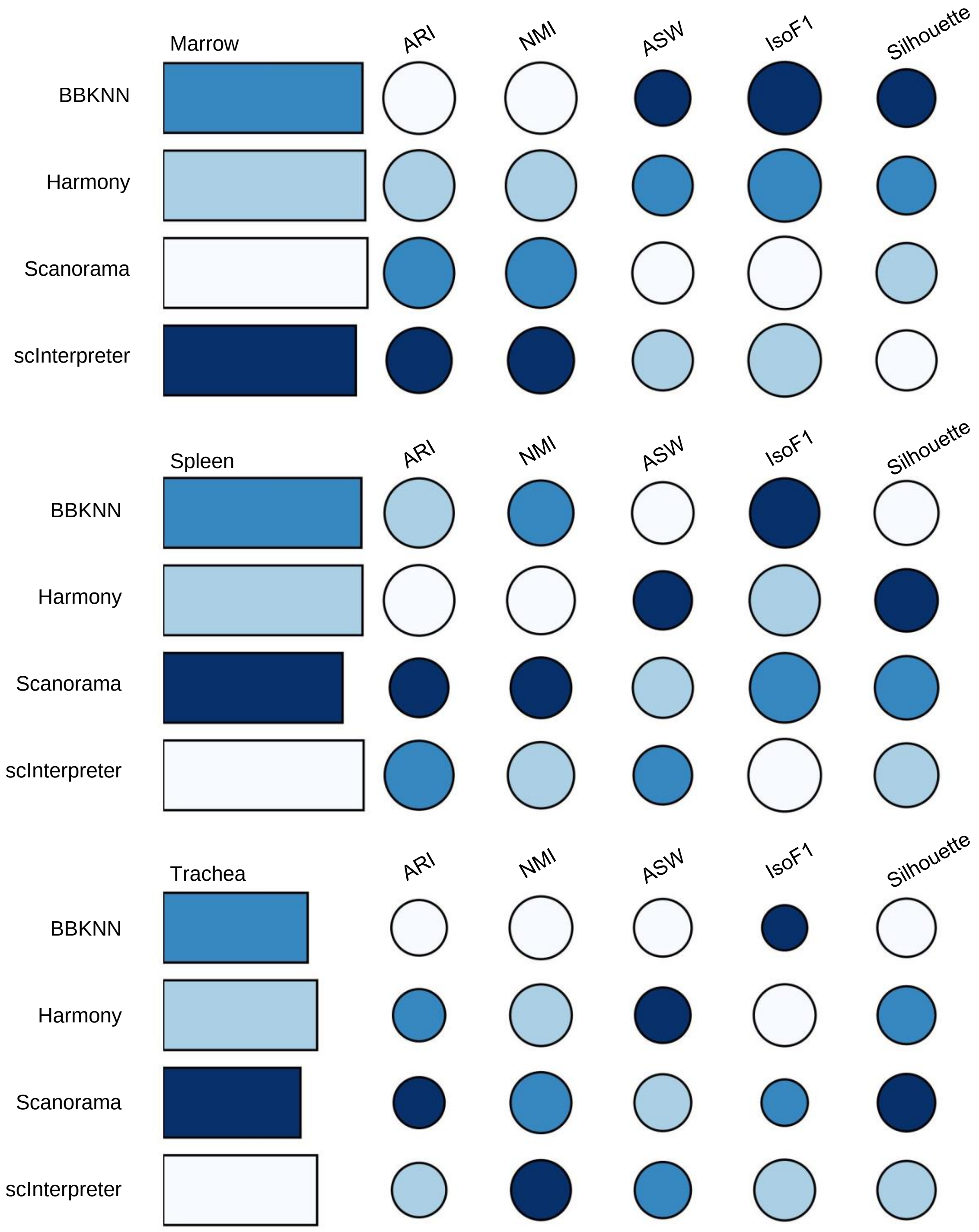

Figure S2, results of scInterpreter and SOTA baselines in multiple tissues of the mouse scRNA-seq atlas

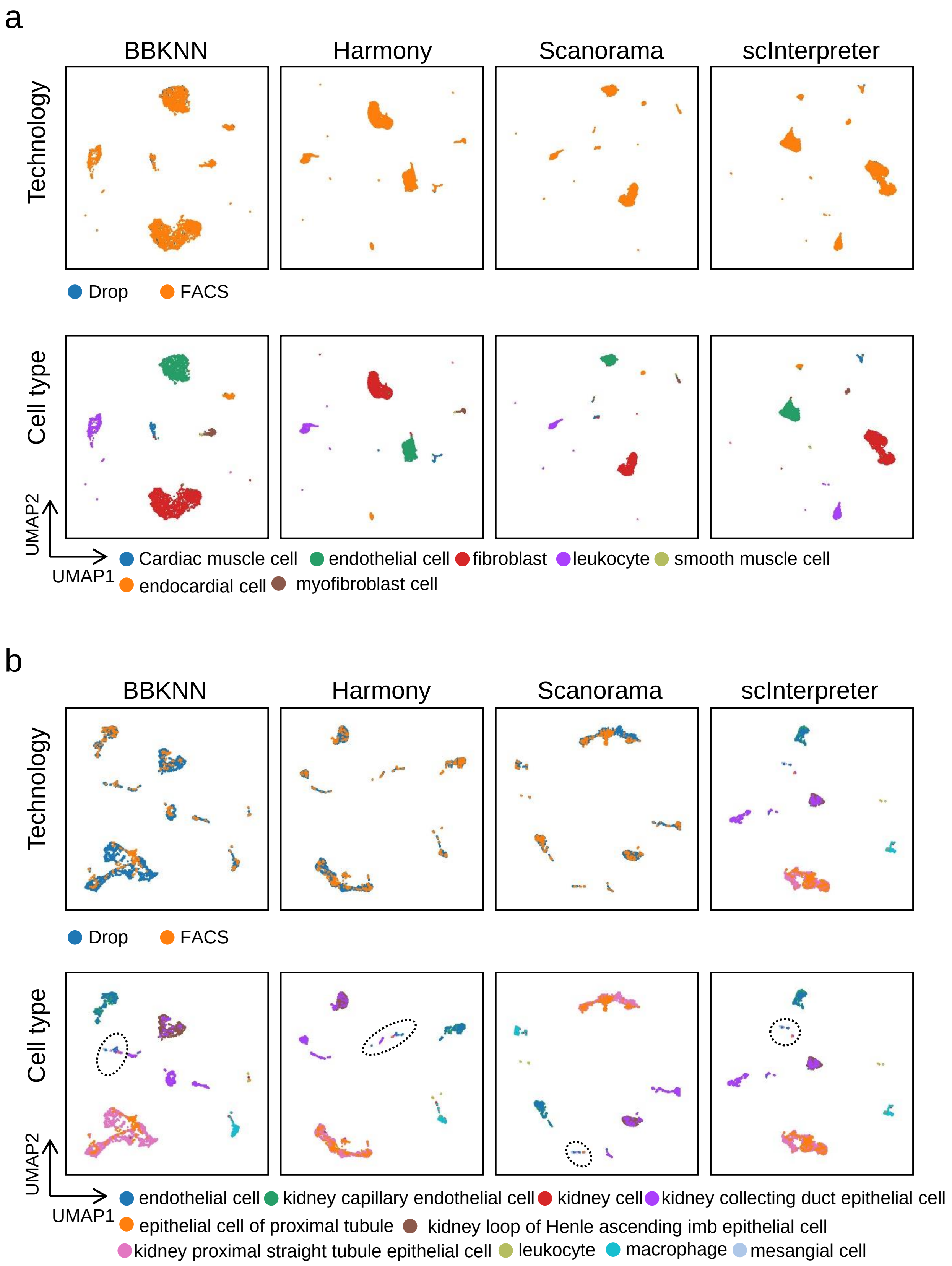

Figure S3 a, UMAP embeddings of the integration results based on the mouse heart scRNA-seq. Cells are colored by the batch (the first row) and cell type (the second row). b, UMAP embeddings of the integration results based on the mouse kidney scRNA-seq. Cells are colored by the batch (the first row) and cell type (the second row).

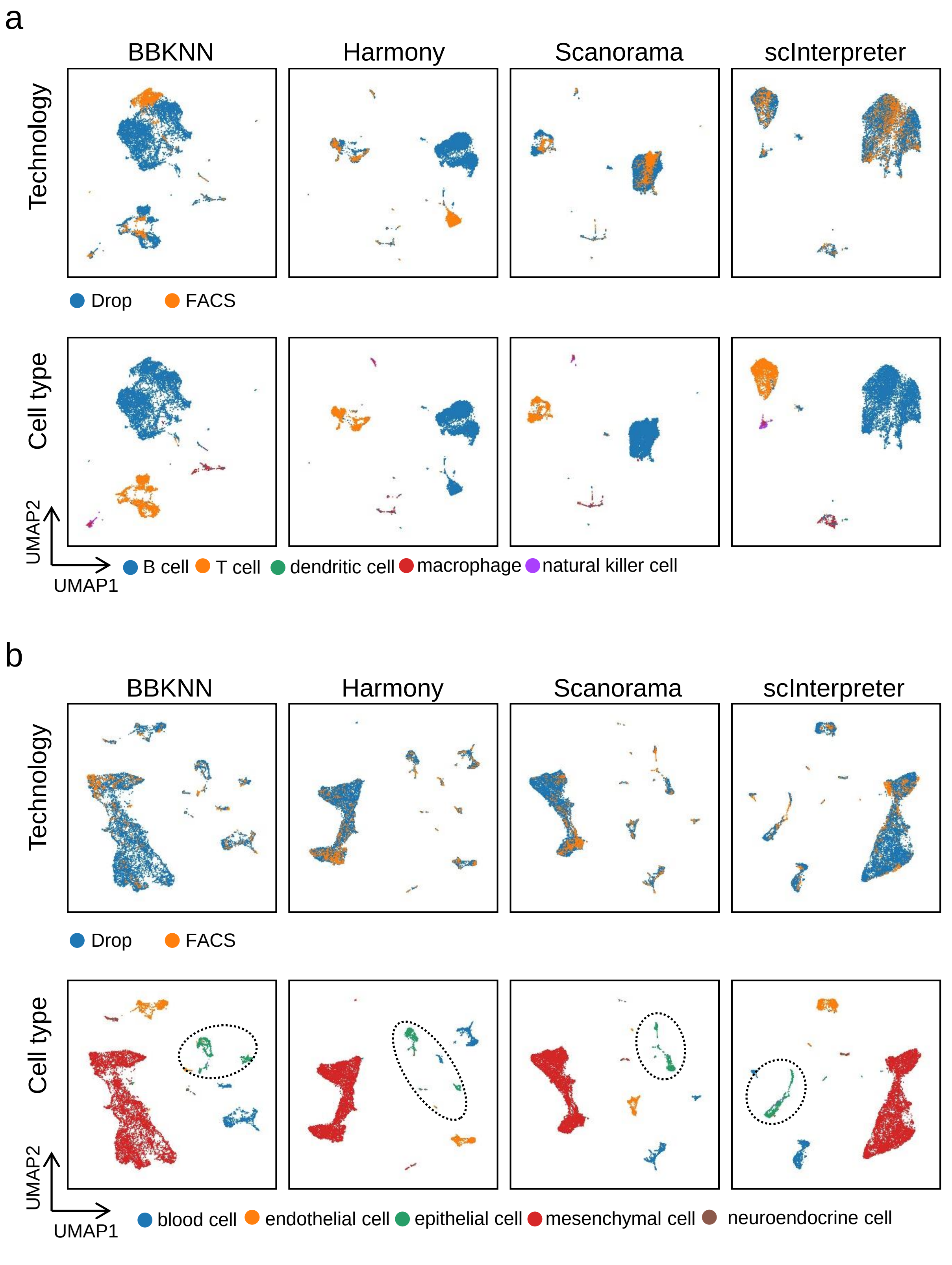

Figure S4 a, UMAP embeddings of the integration results based on the mouse spleen scRNA-seq. Cells are colored by the batch (the first row) and cell type (the second row). b, UMAP embeddings of the integration results based on the mouse trachea scRNA-seq. Cells are colored by the batch (the first row) and cell type (the second row).

a

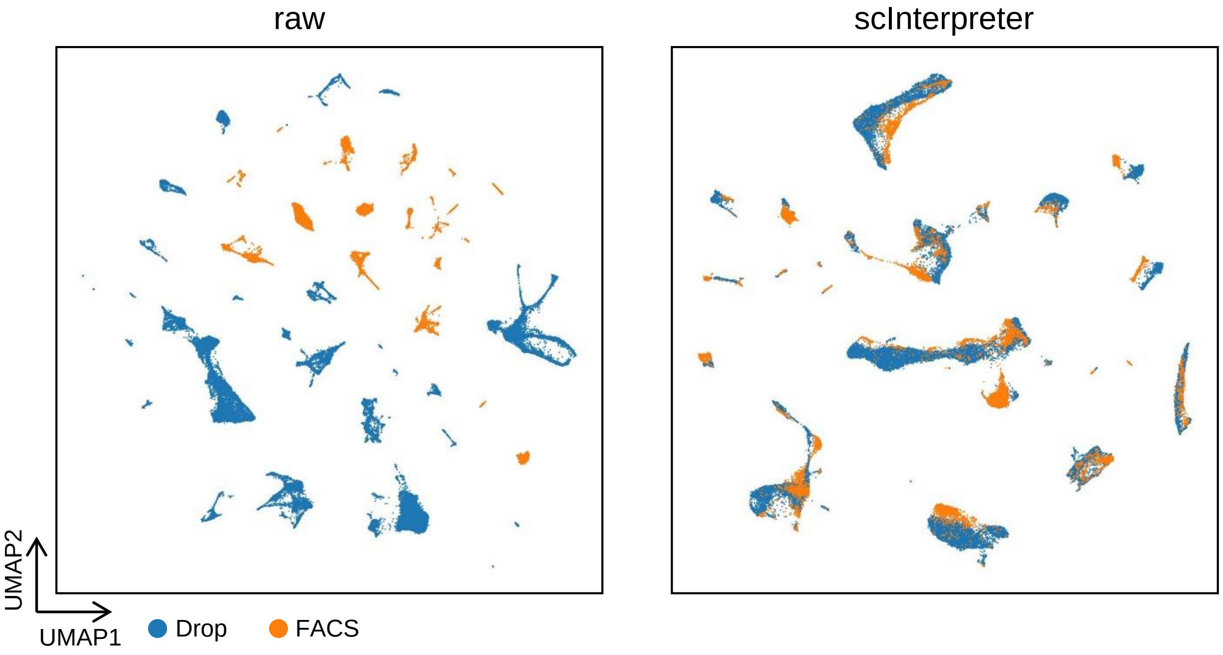

Figure S5 a, UMAP embeddings of the integration results by the raw and scInterpreter based on the mouse atlas. Cells are colored by the batch (the first row) and cell type (the second row).

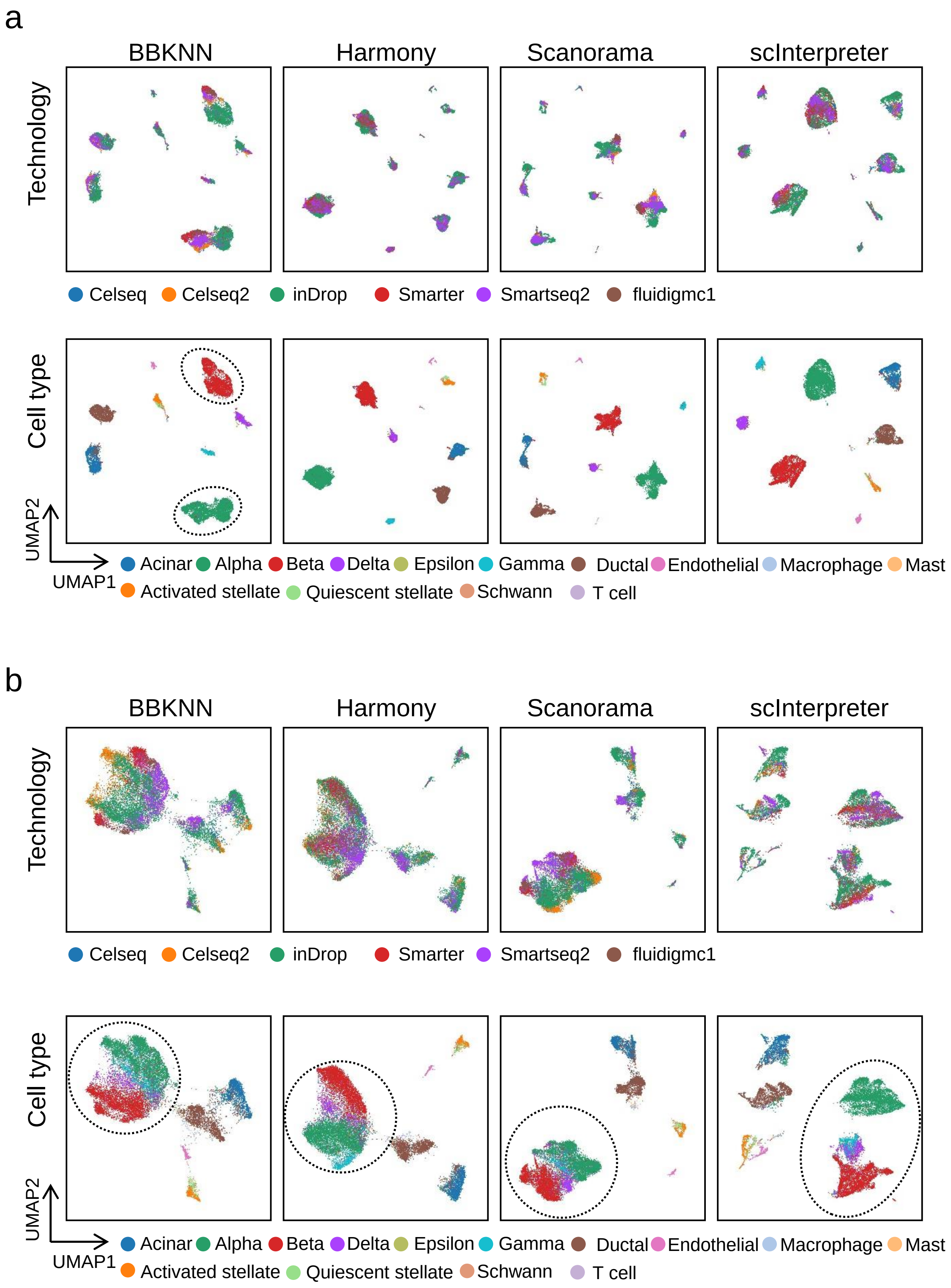

Figure S6 a, We visualized the integration results based on human pancreas dataset. Cells are colored by the batch (the first row) and cell type (the second row). b, UMAP plots of sampling counts by scInterpreter based on human pancreas dataset. Cells are colored by the batch (the first row) and cell type (the second row).

a

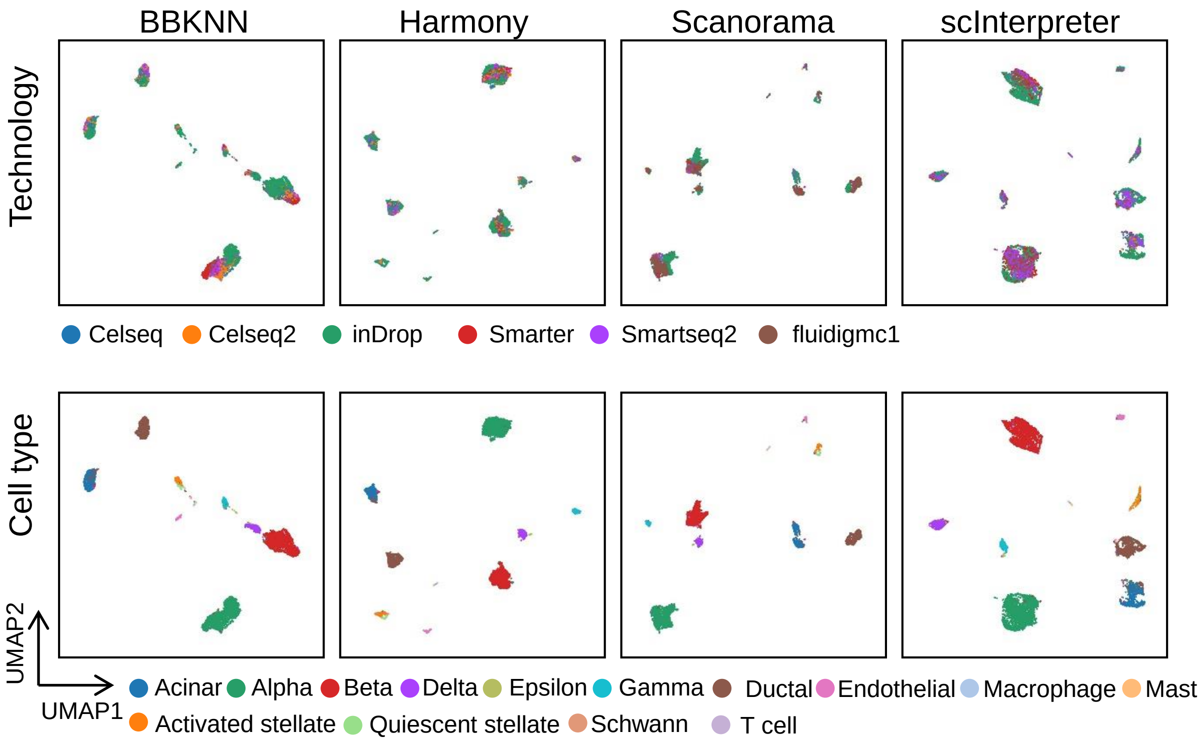

b

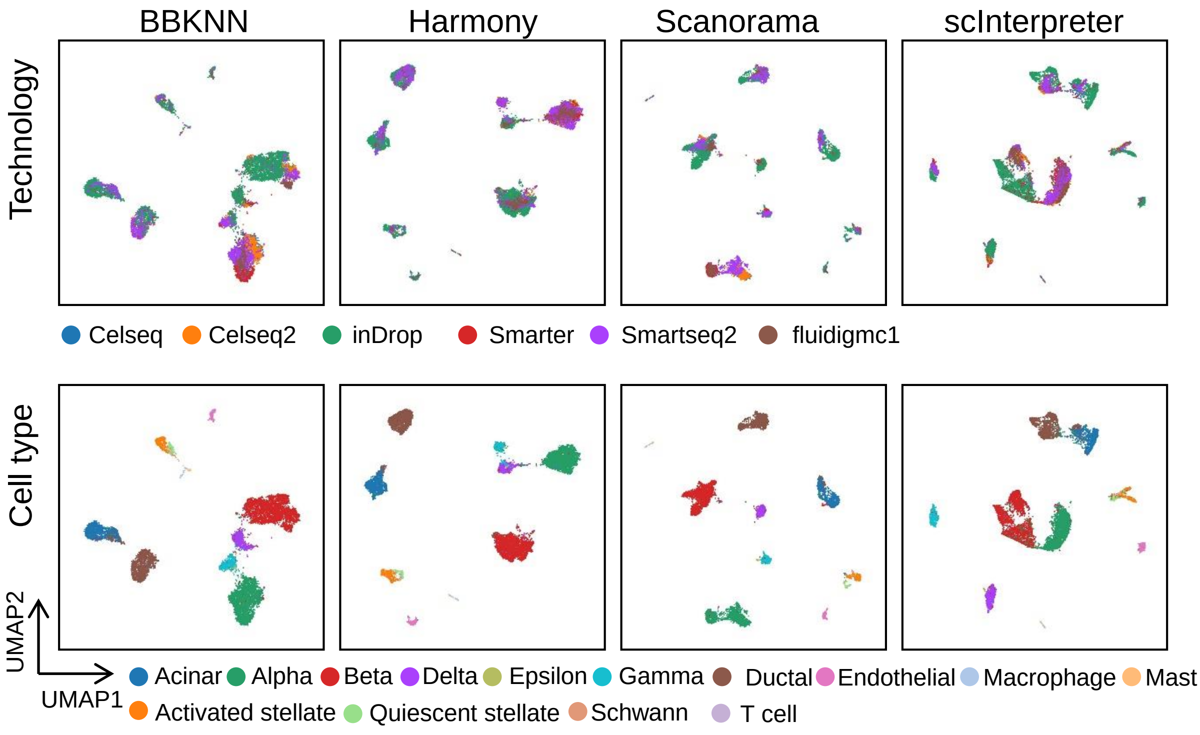

Figure S7 a, UMAP plots of sampling cells by scInterpreter based on human pancreas dataset. Cells are colored by the batch (the first row) and cell type (the second row). b, UMAP plots of sampling cell types by scInterpreter based on human pancreas dataset. Cells are colored by the batch (the first row) and cell type (the second row).

a

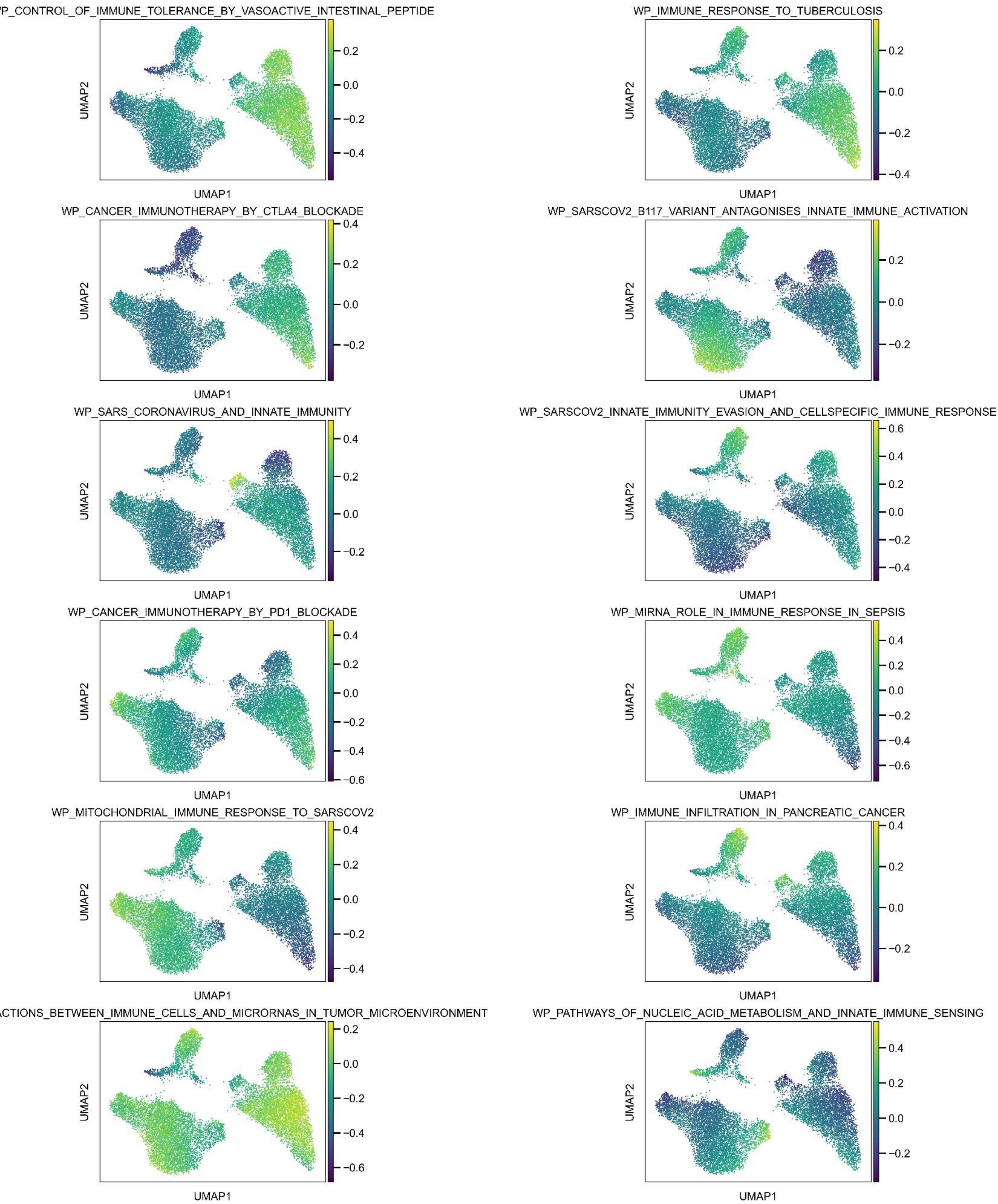

Figure S8 a, UMAP plots of all Immune pathway expression in wiki database on PBMC.

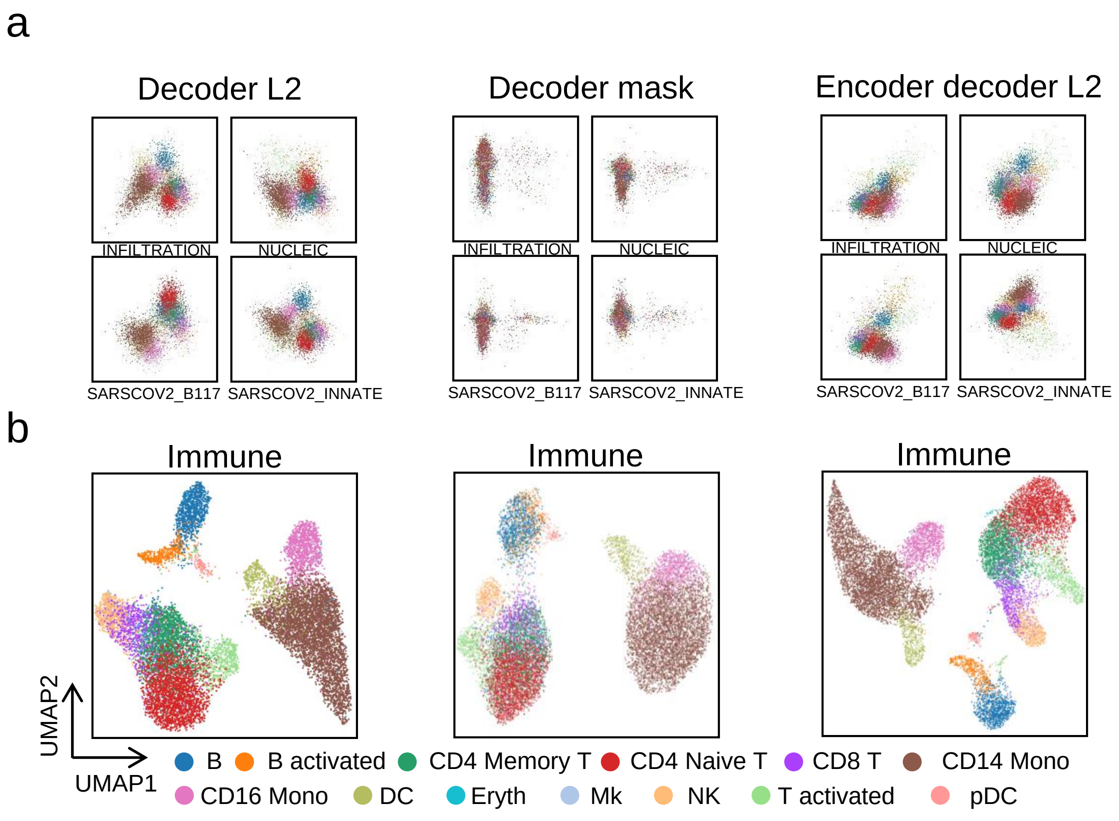

Figure S9 a. Plots of cells in 2 dimension of Immune-related pathway. b. We visualized the integration results from scInterpreter. Cells are colored by cell type.

Table S1, Training time of each method

	Epoch1	Epoch2	Epoch4	Epoch8	Epoch16	Finished training
WIKI	4.98	9.24	20.9	33.9	70.46	
None	4.91	9.34	16.97	33.9	63.49	
BBKNN						1.65
Harmony						41.97
Scanorama						23.7

Time Unit: seconds

Table S2, The highest weights in the decoder

	highly_variable	means	dispersions	dispersions_norm	highly_variable_nbatches	highly_variable_intersection	weight
PLAUR	TRUE	1.494255	2.570846	0.633738	2	TRUE	0.054362
MS4A6A	TRUE	0.712872	2.25591	0.388415	1	FALSE	0.054464
SAT1	TRUE	3.194782	3.800021	0.915767	2	TRUE	0.054704
IFIT3	TRUE	1.893825	2.827376	1.237664	1	FALSE	0.055468
CA2	TRUE	0.389901	2.13739	0.450257	2	TRUE	0.056834
RABGAP1L	TRUE	0.816799	2.119737	0.181437	1	FALSE	0.060188
IFIT2	TRUE	1.60469	3.046135	1.90712	1	FALSE	0.064941
FPR1	TRUE	0.416121	2.048864	0.214066	1	FALSE	0.065709
HIST1H2AC	TRUE	1.106629	2.662483	1.210875	2	TRUE	0.066943
IDS	TRUE	0.602982	2.113432	0.179351	1	FALSE	0.06721

Table S3, The highest absolute weights in the decoder

	highly_variable	means	dispersions	dispersions_norm	highly_variable_nbatches	highly_variable_intersection	weight
TMEM109	TRUE	0.392356	2.16182	0.547482	2	TRUE	-0.05967
TMEM261	TRUE	0.421271	2.1276	0.415707	2	TRUE	-0.05998
RABGAP1L	TRUE	0.816799	2.119737	0.181437	1	FALSE	0.060188
NCL	TRUE	0.78624	2.207886	0.368504	2	TRUE	-0.06087
ESF1	TRUE	0.252883	2.014213	0.298169	1	FALSE	-0.06103
MNAT1	TRUE	0.174292	2.058661	0.451718	2	TRUE	-0.06142
IFIT2	TRUE	1.60469	3.046135	1.90712	1	FALSE	0.064941
FPR1	TRUE	0.416121	2.048864	0.214066	1	FALSE	0.065709
HIST1H2AC	TRUE	1.106629	2.662483	1.210875	2	TRUE	0.066943
IDS	TRUE	0.602982	2.113432	0.179351	1	FALSE	0.06721

Table S4, The dataset used in this study

Datasets	Batche ID	Study (Accession) ID	Species	omics
mouse atlas	Drop	Single-cell RNA-seq data from microfluidic emulsion (v2) (figshare.com)	Mouse	scRNA-seq
	FACS	Single-cell RNA-seq data from Smart-seq2 sequencing of FACS sorted cells (v2) (figshare.com)	Mouse	scRNA-seq
pancreas	indrop1	GSE84133	human	scRNA-seq
	indrop2	GSE84133	human	scRNA-seq
	indrop3	GSE84133	human	scRNA-seq
	indrop4	GSE84133	human	scRNA-seq
	celseq	GSE81076	human	scRNA-seq
	celseq2	GSE85241	human	scRNA-seq
	smart	GSE81608	human	scRNA-seq
	smartseq2	E-MTAB-5061	human	scRNA-seq
	fluidigmc1	GSE86469	human	scRNA-seq
PBMC	Ctrl	https://seurat.nygenome.org/src/contrib/ifnb.SeuratData_3.0.0.tar.gz	human	scRNA-seq
	Stim	https://seurat.nygenome.org/src/contrib/ifnb.SeuratData_3.0.0.tar.gz	human	scRNA-seq
